# Chemoprophylaxis of Tropical Infectious Diseases

**DOI:** 10.3390/ph3051561

**Published:** 2010-05-18

**Authors:** William J. H. McBride

**Affiliations:** School of Medicine and Dentistry, James Cook University, Cairns Base Hospital campus, The Esplanade, Cairns, Queensland 4870, Australia; E-Mail: john.mcbride@jcu.edu.au; Tel.: +617-40506530; Fax: +617-40506831

**Keywords:** chemoprophylaxis, malaria, leptospirosis, scrub typhus, diarrhea

## Abstract

Travelers to tropical countries are at risk for a variety of infectious diseases. In some cases effective vaccinations are available, but for other infections chemoprophylaxis can be offered. Malaria prevention has become increasingly complex as *Plasmodium* species become resistant to available drugs. In certain high risk settings, antibiotics can be used to prevent leptospirosis, scrub typhus and other infections. Post-exposure prophylaxis is appropriate for selected virulent infections. In this article the evidence for chemoprophylaxis will be reviewed.

## 1. Introduction

Chemoprophylaxis is the administration of drug to prevent the development of a disease. This review will focus on the use of medications to prevent tropical infectious diseases. The use of chemo-prophylactic agents is based on knowledge of the epidemiology and clinical implications of the infectious diseases from which protection is sought. Generally, chemoprophylaxis is taken for diseases that are common, or where the clinical impact of infection is high.

Drugs may be taken before exposure (pre-exposure prophylaxis) or after potential exposure to an infectious agent (post-exposure prophylaxis). In addition to the severity and frequency of the disease, the tolerability, toxicity and ecological implications of the medications being used are important considerations in whether drugs are prescribed and taken. In many instances, vaccines are the most effective and safest means by which to prevent infection. This review will not discuss vaccination as this is well covered elsewhere [1]. Passive immunotherapy is also used to prevent infection either before or after potential exposure to infectious agents. Again this review will not discuss this form of therapy in detail. Immunotherapy is also reviewed elsewhere [2,3]. This review will discuss chemoprophylaxis for specific infections and disease syndromes in the order of how widely used and acceptable their use is. A summary of recommendations is shown in Table 1.

**Table 1:** Recommended drugs for use in chemoprophylaxis of tropical infections.

	**Infection/Patient Group**		**Recommended Drug**	**Comments**
**Pre-exposure Prophylaxis**	Malaria	Adult—Short visit(less than 1 week)	Atovaquone/proguanil (Malarone), Primaquine, or any of the drugs listed below.	These 2 drugs are taken daily for 2 days prior to travel, during, and for 7 days post travel. Check G6PD status for primaquine.
		Adult—Long visit(more than 1 week)	Doxycycline, Mefloquine, other drugs listed above.	Mefloquine taken weekly for 2 weeks prior to travel. Doxycycline is taken daily for 2 days prior to travel. Both drugs are taken for the duration of travel and for 4 weeks after return. Mefloquine resistance in some parts of southeast Asia.
		Pregnant women	Mefloquine, Chloroquine+proguanil	Chloroquine resistance widespread, combination of Chloroquine + proguanil less well tolerated than comparators.
		Children	Mefloquine, Primaquine, Malarone (>5 kg), Doxycycline (>8 years)	Check G6PD status for primaquine.
	Leptospirosis		Doxycycline, 200 mg weekly	May only act to reduce clinical illness rather than infection. May cause nausea and vomiting.
	Scrub typhus		Doxycycline, 200 mg weekly	May only act to reduce clinical illness rather than infection. May cause nausea and vomiting.
	Traveller’s Diarrhoea		Rifaximin, Quinolones (Norfloxacin, Ciprofloxacin)	Rifaximin prophylactic efficacy shown in Mexico, Quinolones best reserved for presumptive treatment rather than prophylaxis.
	Schistosomiasis		Artemether	Only for unavoidable high risk exposure
	Leprosy		Rifampicin	Only for unavoidable high risk exposure
	HIV		Tenofovir	Unproven
**Post-exposure Prophylaxis**	Burkholderia pseudomallei (Melioidosis)		Co-trimoxazole or Doxycycline	Usually for known laboratory exposures
	Brucella spp (Brucellosis)		Doxycycline + Rifampicin or Co-trimoxazole	Usually for known laboratory exposures
	Yersinia pestis (Plague)		Doxycycline or Ciprofloxacin	
	Bacillus anthracis (Anthrax)		Doxycycline or Ciprofloxacin	
	Cercopithecine herpesvirus 1 (Herpes B virus)		Valacyclovir	Consider for percutaneous exposure (bites) from asian macaque monkeys

## 2. Malaria

Malaria is blood protozoan parasite which is transmitted by mosquitos. There are four human species: *Plasmodium falciparum*, *Plasmodium vivax*, *Plasmodium ovale* and *Plasmodium malariae* [4]. A fifth species, *Plasmodium knowlesi* is also a parasite of several macaque species, and is thus zoonotic. It is a common cause of malaria in Malaysian Borneo, but is also found on the Malay Peninsula, Singapore, the Philippines, Thailand and Myanmar [5,6,7,8,9]. 

Malaria is endemic in 109 countries and causes an estimated 247 million infections worldwide and about 1 million deaths [10]. Of the estimated 50 million international travelers per year, between 1–5% are ill enough on return to seek medical attention, and up to 3% of short term travelers report fevers [11]. Malaria is the most common cause of fever in returned travelers, but this is strongly related to the travel history. Fever in a traveler returning from Sub-Saharan Africa is almost exclusively due to malaria, whereas visitors to the Caribbean and South East Asia are more likely to have dengue fever than malaria as a cause of their fever [12].

From 1997 through 2006, 10,745 cases of malaria among U.S. residents were reported to CDC. During this period, 54 fatal malaria infections occurred among U.S. residents; 46 (85.2%) were caused by *P. falciparum*, of which 33 (71.1%) were acquired in sub-Saharan Africa [1]. Drugs used to prevent malaria include chloroquine, hydroxychloroquine, atovaquone/proguanil (Malarone^®^), doxycycline, mefloquine, primaquine and tafenoquine. None of the drugs are totally effective at preventing malaria and should always be given in conjunction with general advice on avoidance of mosquito bites through behavioral means, protective clothing and insect repellants.

### 2.1. Chloroquine and Hydroxychloroquine

Chloroquine (CQ) is a 4-aminoquinolone that accumulates in the parasitic food vacuole, raising the pH of the vacuole, which interferes without the production of hemozoin, leading to accumulation of toxic haem metabolites. The drugs acts on the erythrocytic stage of the life cycle but is not active against gametocytes, sporozoites, the hepatic schizont or hypnozoite stage of the life cycle. Resistance to chloroquine is widespread amongst *P. falciparum* strains and is the result of increased efflux of the drug from the food vacuole [13]. More recently *P. vivax* strains have been increasingly recognized, initially in Papua New Guinea, but there are reports of resistance now in Asia and South America [14]. Chloroquine has high oral bioavailability of 90%, and a long half life of four days, making weekly dosing possible for prophylaxis. Given over a long period, chloroquine may cause retinal toxicity [13]. The drug is considered safe in pregnancy and is still effective in preventing *P. vivax* where *P. vivax* is common [15]. Hydroxychloroquine has less retinal toxicity and has equivalent activity to CQ against CQ sensitive strains, but is considerably less active than CQ against CQ resistant strains of *P. falciparum* [16].

### 2.2. Atovaquone/Proguanil (Malarone^®^)

Atovaquone is a hydroxynapthoquinone with activity against all species of *Plasmodium*, *Pneumocystis jirovecii*, and *Toxoplasma gondii*. It blocks electron transport at the level of the cytochrome bc_1_ complex. Pyrimidine synthesis is coupled to this electron transport system. Mammalian and parasite electron transport systems are differentially sensitive to the effects of atovaquone. Used as monotherapy in preclinical studies there was a high level of recrudescent malaria. Proguanil is a dihydrofolate reductase inhibitor and acts synergistically with atovaquone in inhibiting pyrimidine synthesis. The combination of the two drugs was highly efficacious in the treatment of *P. falciparum* [13,17]. Malarone^®^ is taken daily, but because of its’ activity on the hepatic schizont stage of the life cycle it is considered causal prophylaxis and only needs to be taken for one week after leaving a malarial area [4]. Recent meta-analyses of the efficacy of Malarone^®^ used as prophylaxis has demonstrated protective efficacy of 95.8% compared to placebo [18]. Malarone^®^ is very well tolerated and is safe in children weighing over 5 kg. In adults, tolerability is comparable with placebo, but in higher doses used for treatment, gastrointestinal intolerance is more common [19]. It is not recommended for use in pregnancy [20,21]. There is a single case report of Stevens-Johnson syndrome in association with Malarone^®^ [22]. 

### 2.3. Mefloquine

Mefloquine is a 4-quinolonemethanol related to quinine and chloroquine, and is similarly active against the same stages of the life cycle of *Plasmodium spp*. It is, however, active against chloroquine resistant strains of malaria. It is highly orally bioavailable and protein bound with a half life of 14–28 days. Given weekly it reaches steady state concentration in about 10 weeks. The drug should be started 1–2 weeks prior to entry into a malarial area and continued for four weeks after return. A rare, but serious side effect is neurotoxicity, and is more common if mefloquine is used to treat severe malaria [13]. Mefloquine is contraindicated in patients with depression and a history of psychiatric disturbance. On the other hand, mefloquine is the only drug, apart from chloroquine, considered safe for use in pregnancy. It can also be used in children [1]. Compared to Malarone^®^, mefloquine had slightly higher rates of gastrointestinal and neuropsychiatric side effects [4]. Mefloquine resistance occurs near the borders between Thailand, Laos, China and Myanmar, western Cambodia and central Vietnam [1]. 

### 2.4. Doxycycline

Doxycycline is thought to act on the plastid-like organelle of *Plasmodium spp* called the apicoplast, which harbors prokaryotic-type biochemical pathways [23]. Tetracyclines bind to the 30S ribosomal subunit and inhibit protein synthesis. Doxycycline is well absorbed orally and has a half-life of 15–24 hours. Resistance has not yet been reported in protozoa. Photosensitivity is the most problematic toxicity in travelers, although in a blinded study comparing doxycycline with other antimalarials, skin reactions were no more frequent in those taking this drug [24]. Doxycycline is not recommended in pregnant women and children less than 8 years because of the potential for enamel hypoplasia causing tooth discoloration [25]. Doxycycline is taken daily for 1–2 days prior to travel to malarial areas and continued for 4 weeks after travel [20]. Comparisons with mefloquine have shown that doxycycline confers comparably high rates of protection against malaria (99–100%), but that compliance is less with the daily doxycycline than with weekly mefloquine (75% *vs.* 100%) [26,27]. There were less neuropsychiatric adverse events with doxycycline [4].

### 2.5. Primaquine

Primaquine is an 8-aminoquinolone, and its mode of action is not well understood. It may interfere with ubiquinone function in parasite mitochondria, or generate high oxidative stress within the parasite [28]. The high oxidative stress caused by the drug is the reason for the requirement to verify that patients taking the drug are not deficient in glucose-6-phosphate dehydrogenase (G6PD). Primaquine is well absorbed orally and has a short half life, however it is prodrug and the pharmcokinetics of its metabolites, which are responsible for its activity, are not well understood. In non-G6PD deficient patients the drug is generally well tolerated, although some have gastrointestinal symptoms. It is considered safe in children but is contraindicated in pregnancy. As chemoprophylaxis, primaquine can be used as terminal prophylaxis or primary prophylaxis. It's most common use in malaria however, is in the treatment of *P. vivax* to prevent recurrences. Primaquine is active against all exoerythocytic stages of the malaria life cycle, including unique activity against hepatic hypnozoites. Primaquine’s activity against hepatic trophozoites provides causal prophylaxis and recommended duration of therapy is from two days before, to one week after, visits to malarial areas. Protective efficacy at a dose of 30 mg/day in adults has ranged from 88–94.5% [29,30]. Terminal prophylaxis is sometimes given to people who have not taken causal prophylaxis and who are at particularly high risk of having acquired malaria (e.g., soldiers deployed to malaria endemic areas) [31]. Radical cure of *P. vivax* is required after documented infection with this parasite to prevent relapse. The dose of primaquine for both terminal prophylaxis and radical cure is 30 mg/day in adults. Previously recommended lower doses were associated with unacceptable relapse rates [30,32]. An additional use of primaquine is the prevention of transmission to mosquitoes, taking advantage of its’ gametocidal properties [33].

### 2.6. Tafenoquine

Tafenoquine is an experimental, long acting 8-aminoquinolone. The risk of hemolysis in G6PD deficient people also exists with this drug, however weekly administration is seen as a major advantage. Several studies have demonstrated efficacy of 86–89%, or equivalence with mefloquine in non-inferiority studies [34,35,36]. Corneal deposits (vortex keratopathy) were observed in a high proportion of subjects taking tafenoquine but these deposits spontaneously resolve after cessation of administration and did not affect vision [35].

### 2.7. Choice of Malaria Chemoprophylaxis

There are many factors that influence the choice of chemoprophylaxis agent (see Table). Long term residents of malaria endemic countries may choose not to take prophylaxis, take drugs seasonally, when exposed to especially high risk, or to take presumptive treatment when unwell. None of the medications are free of adverse effects. Rates of adverse events are comparable to placebo with doxycycline and malarone, slightly higher with mefloquine and higher again with combined chloroquine and proguanil [24]. Weekly prophylaxis regimens are more convenient for longer durations [37]. For short trips, agents that offer causal prophylaxis (Malarone^®^, primaquine) might be preferred because of the need to continue these medications for one week instead of four after return. Both malarone and mefloquine can be used in children. Mefloquine is the preferred agent in pregnancy, although the combination of chloroquine and proguanil is sometimes recommended [38]. In 2007 patients surveyed in Switzerland used mefloquine at about twice the rate of Malarone^®^, and doxycycline was slightly less used than Malarone^®^ [39]. More recently Malarone^®^ use is growing in popularity at the expense of mefloquine [40]. Cost may influence choice of agent, with Malarone^®^ often being most expensive.

## 3. Traveler’s Diarrhea

Diarrhea is the most common affliction for travelers to tropical countries. Between 20–60% of visitors to resource poor countries are affected [12,41]. Prevention is primarily through attention to food and hand hygiene. Vaccination is available for cholera, typhoid, entertoxigenic *E. coli* (ETEC), and rotavirus. Chemoprophylaxis, using rifaximin has been shown to be effective in reducing the incidence of traveler’s diarrhea [42]. Rifaximin is a semi synthetic derivative of rifamycin. Taken, orally it is not absorbed and achieves high concentrations intraluminally [43]. In a dose of 200 mg/day it reduced traveler’s diarrhea by 72% [44]. It is considered safe in children more than 2 years old but not in pregnancy. Quinolone antibiotics (ciprofloxacin and norfloxacin) are around 90% efficacious in preventing traveler’s diarrhea, but are associated with development of resistant organisms, are not generally recommended as chemoprophylaxis. They do have a role in presumptive treatment of diarrhea episodes [45]. Chemoprophylaxis for traveler’s diarrhea should not be routinely provided but may be useful in circumstances where diarrheal episodes could prove inconvenient or clinically significant for the traveler [43]. Doxycycline was shown to reduce episodes of diarrhea in Peace Corps volunteers in Honduras, however diarrhea episodes in soldiers in Thailand, receiving either mefloquine or doxycycline occurred in nearly 50% with either drug. Both were associated with increased antibiotic resistance [46,47]. There is no evidence to support the choice of an antimalarial drug in the prevention of traveler’s diarrhea. 

## 4. Scrub Typhus

Scrub typhus is caused by *Orientia tsutsugamushi* and transmitted to humans via the bite of the mite, *Leptotrombidium diliense* [48]. Cases of scrub typhus tend to geographically localized and clusters of cases can occur when groups of people are exposed, through close contact with vegetation, in these areas [49]. These exposures can sometimes be predicted and use of doxycycline in a dose of 200 mg weekly has been shown to be effective in field and laboratory experiments at preventing disease, but not infection [50,51]. Azithromycin is effective in the treatment of scrub typhus but there is no evidence to support its’ use in chemoprophylaxis [52,53,54].

## 5. Leptospirosis

Leptospirosis is a zoonotic bacterial disease transmitted to humans who come in contact with water that has been contaminated with the urine of infected animals. Certain groups of people, e.g., farmers, or participants in water sporting events, are at higher risk for acquisition of this disease, and floods may be associated with large outbreaks [55,56,57,58,59,60,61]. Treatment of leptospirosis is with penicillin, cefotaxime or doxycycline, which are equally effective [62]. Azithromycin has been shown to be also effective in the treatment of humans and in a lethal guinea pig model [54,63]. Penicillin given as post-exposure prophylaxis has been unsuccessful [64]. The efficacy of doxycycline for chemoprophylaxis of leptospirosis has not been consistent in all trials. A study of US soldiers training in Panama demonstrated a 95% protective efficacy with a weekly dose of 200 mg doxycycline, compared to placebo [65]. Two later placebo controlled studies have shown that although doxycycline modestly reduces the rate of clinical disease, it does not reduce the infection rate [66,67]. A recent Cochrane review highlights that doxycycline side effects of nausea and vomiting should be balanced against the relatively weak evidence of prophylactic efficacy [68].

## 6. Pre-Exposure Chemoprophylaxis of Other Tropical Infections

Schistosomiasis is a common problem in returned travelers, especially after exposure to freshwater in Africa (e.g., Lake Malawi). Longer term travelers, such as missionaries, volunteers and researchers have double the risk of infection [69,70]. Artemether, a methyl ether derivative of quinghaosu, has anti-schistosomal properties, and because it affects the parasite at the larval stage was proposed as a chemo prophylactic agent for this disease [71]. A randomized double blinded placebo controlled study in African children after radical treatment demonstrated a 50% reduction in new infection in children who received a course of artemether every three weeks. Not surprisingly malaria prevalence was also reduced [72]. Chemoprophylaxis against schistosomiasis should only be considered for unavoidable exposure in high risk settings.

Leprosy is a skin and nerve disease caused by *Mycobacterium leprae*. There are a number of studies now showing that single doses of rifampicin are effective at a population level and amongst close contacts (post-exposure) in preventing leprosy [73,74,75,76,77]. Chemoprophylaxis for leprosy would normally only be considered in special circumstances in travelers, but has a role to play in indigenous populations of endemic areas. 

Exposure to HIV is a risk for sexually active people, especially in high prevalence countries. Chemoprophylaxis, using the anti-retroviral drug, tenofovir disoproxil fumarate, has been attempted in women who were HIV negative, but at risk of acquiring HIV. Women took 300 mg of tenofovir daily, and although the infection rate appeared to be lower than in the placebo group, the study finished prematurely and the results were not statistically significant [78,79]. Use of chemoprophylaxis for this indication is not justified currently on the basis of the evidence available, and would only be applicable to people who were powerless to protect themselves in other proven ways, such as the use of condoms.

## 7. Post-Exposure Chemoprophylaxis for Tropical Infections

There are several infectious diseases whose occurrence is uncommon but where an infecting event can be identified and preventative chemotherapy is warranted. Laboratory workers can be accidentally exposed to pathogens that are more common in the tropical environments and imported by travelers who have returned from these environments. *Burkholderia pseudomallei* is a gram negative organism that can cause a multisystem septic illness called melioidosis. People with underlying medical conditions such as diabetes are particularly at risk [80]. In mouse model of infection, co-trimoxazole was found to offer the greatest protection both before, and within 24 hours of exposure. Amoxicillin/clavulanic acid was the least active agent tested in this model [81]. Co-trimoxazole and doxycycline has been used for 3 weeks as post-exposure prophylaxis after laboratory exposure to *Burkholderia pseudomallei*, although adverse reactions to this drug can be a problem [82,83]. Brucellosis is a bacterial infection caused by one of several species of *Brucella*. This organism is notoriously infectious, unless cultures are manipulated in biosafety hoods. Laboratory workers exposed to this organism are usually given a combination of doxycycline and rifampicin for 3 weeks or co-trimoxazole [84,85]. *Yersinia pestis*, the causative agent of plague, is an extremely rare cause of infection in the modern era, however sporadic cases and outbreaks still occur. The disease is considered a potential bioterrorism threat and chemoprophylaxis with either doxycycline or ciprofloxacin is recommended after exposure [86]. Quinolone antibiotics and doxycycline are similarly recommended as post-exposure prophylaxis for *Bacillus anthracis*, the causative agent of anthrax [87].

Hendra virus infection is a risk for veterinarians and people who come into contact with sick horses in Australia. On the basis of data that ribavarin, a broad spectrum antiviral, was associated with favorable outcomes in nipah virus infections, the use of this drug has been used as post exposure prophylaxis. The combination of ribavarin and chloroquine was not, however, effective in an *in vivo* hamster model of infection [88,89,90].

Herpes B virus (Cercopithecine herpesvirus 1) is carried by over 80% of Asian macaque monkeys, and there are rare reports of fatal encephalomyelitis in humans after percutaneous exposures. Valacyclovir is recommended within 24 hours after high risk exposures [91,92,93].

Post-exposure prophylaxis is used for infections such as Influenza, *Neisseria meningitidis*, *Haemophilus influenza* type B, and *Bordatella pertussis*. They are not discussed as they are not considered to be predominantly tropical.

## 8. Conclusions

Prophylaxis of an infection with an anti-infective is theoretically possible for any infection that has a satisfactory treatment. Pre-exposure prophylaxis is effective when the risk of acquiring infection is high and the drugs used to prevent the infection are well tolerated and easy to take. This is generally the case for malaria prophylaxis taken by travelers. Chemoprophylaxis for other tropical infections is not as well established but should be considered for the traveler who plans to expose themselves to a higher level of risk. Post-exposure prophylaxis is provided after unforeseen exposure to infectious agents that can have serious consequences. In this situation the drug regimens do not necessarily need to be as convenient, or well tolerated, as medication taken for pre-exposure prophylaxis. In many cases chemoprophylaxis should play a secondary role to other non-drug measures that are known to reduce the risk of infection.
